# The mTOR Inhibitor Rapamycin Synergizes with a Fatty Acid Synthase Inhibitor to Induce Cytotoxicity in ER/HER2-Positive Breast Cancer Cells

**DOI:** 10.1371/journal.pone.0097697

**Published:** 2014-05-27

**Authors:** Chen Yan, Huang Wei, Zheng Minjuan, Xue Yan, Yang Jingyue, Liu Wenchao, Han Sheng

**Affiliations:** 1 Department of Oncology, Xi'jing Hospital, Fourth Military Medical University, Xi'an, PR China; 2 Department of Cardiology, Xi'jing hospital, Fourth military medical university, Xi'an, PR China; 3 Department of Ultrasound, Xi'jing Hospital, Fourth Military Medical University, Xi'an, PR China; 4 State Key Laboratory of Military Stomatology, Department of Information Center, School of Stomatology, Fourth Military Medical University, Xi'an, PR China; Institut für Pathologie, Greifswald, Germany, Germany

## Abstract

Patients with ER/HER2-positive breast cancer have a poor prognosis and are less responsive to selective estrogen receptor modulators; this is presumably due to the crosstalk between ER and HER2. Fatty acid synthase (FASN) is essential for the survival and maintenance of the malignant phenotype of breast cancer cells. An intimate relationship exists between FASN, ER and HER2. We hypothesized that FASN may be the downstream effector underlying ER/HER2 crosstalk through the PI3K/AKT/mTOR pathway in ER/HER2-positive breast cancer. The present study implicated the PI3K/AKT/mTOR pathway in the regulation of FASN expression in ER/HER2-positive breast cancer cells and demonstrated that rapamycin, an mTOR inhibitor, inhibited FASN expression. Cerulenin, a FASN inhibitor, synergized with rapamycin to induce apoptosis and inhibit cell migration and tumorigenesis in ER/HER2-positive breast cancer cells. Our findings suggest that inhibiting the mTOR-FASN axis is a promising new strategy for treating ER/HER2-positive breast cancer.

## Introduction

Breast cancer is the second-leading cause of cancer death and morbidity among women worldwide[1]. Gene expression profiling has revealed that breast cancer is a heterogeneous entity, and four primary molecular subgroups have been proposed: basal-like, luminal A, luminal B and human epidermal growth factor receptor 2 (HER2)-overexpressed[2], [3]. Estrogen receptor (ER)/HER2-positive (ER+/HER2+) breast cancer belongs to the luminal B subtype and accounts for 20–25% of all breast cancer cases [4]. Studies have shown that ER+/HER2+ patients have a poor prognosis. In contrast to ER-positive/HER2-negtive breast cancer, ER+/HER2+ patients are less responsive to selective estrogen receptor modulators (SERMs), such as tamoxifen, and to aromatase inhibitors(AIs) [2], [3], [5].

Recent studies have demonstrated that bidirectional crosstalk between ER and HER2 leads to endocrine resistance in ER+/HER2+ breast cancer [6], [7]. Agents that block HER2, such as trastuzumab (Herceptin®) and lapatinib (Tykerb®),improve the inhibitory effects of SERMs in ER+/HER2+ cancer. However, nearly 50% of ER+/HER2+ patients show no response[4], [8]. Furthermore, primary or acquired resistance to trastuzumab has been recognized as a major obstacle in the treatment of this disease[9], [10]. Several clinical trials have shown that combining HER2 inhibitors with SERMs improved progression-free survival but did not extend overall survival (OS) [11]–[13]. Consequently, there is a significant need for elucidating the molecular signaling pathways that promote ER+/HER2+ breast cancer to enable the development of novel therapeutics. Interfering with the growth factor-driven signaling pathways and downstream effectors involved in ER/HER2 crosstalk may lead to the development of new strategies for the treatment of ER+/HER2+ breast cancer.

Fatty acid synthase (FASN) is the enzyme that is responsible for the cellular synthesis of palmitate. As a metabolic oncogene, FASN is constitutively overexpressed and hyperactive in aggressive breast carcinoma[14], [15]. The up-regulation of FASN in tumors is an early and nearly universal epigenetic change that is involved in the development, maintenance and enhancement of the malignant phenotype[14], [15]. We hypothesized that FASN was the key downstream effector of the bidirectional ER/HER2 crosstalk that promotes malignant phenotypes, such as proliferation, migration, apoptosis evasion and endocrine resistance, in ER+/HER2+ breast cancer cells. There is bidirectional crosstalk between FASN and HER2 in cancer cells[16]–[18].FASN overexpression positively correlates with HER2 amplificationin breast cancer cells. FASN is the downstream mediator of HER2 tumorigenicity and cancer progression. FASN inhibition decreases HER2 expression by up-regulating PEA3, a HER2 transcriptional inhibitor, and by changing the lipid composition and function of tumor cell membranes, thereby altering the cellular localization of HER2. In addition, inhibiting FASN negatively affects the interaction between EGFR and HER2, which is a mechanism of trastuzumab resistance in breast cancer[16]–[18]. FASN is regulated by estrogen in ER-positive breast cancer cells; estrogen stimulates FASN expression. FASN expression is part of the E2-mediatedcellular response that leads to the proliferation of hormone-dependent carcinoma cells[19]–[21]. Inhibiting FASN dramatically augments E2-stimulated, ER-driven transcriptional activity, synergistically enhances the E2-mediated down-regulation of ER expression and impairs E2-induced nuclear accumulation of ER. Furthermore, inhibiting FASN induces antitumor activity by acting as a SERM in ER-positive breast cancer cells[19]–[21].Therefore, FASN is most likely the downstream effector underlying ER/HER2 crosstalk in dual-positive breast cancer, but the signaling pathway that is involved remains unknown.

The mammalian target of rapamycin (mTOR) signaling pathway is one of the most important pathways in signal transduction in cancer. mTOR is a serine/threonine-specific kinase that is responsible for mitogen-induced cell proliferation, survival and motility in cancer cells[22]. The mTOR signaling pathway may connect ER/HER2 crosstalk with the downstream effector FASN. HER2amplification activates the mTOR signaling pathway[23]. Inhibiting mTOR blocks multiple stages of HER2-induced tumorigenic progression and improves the antitumor activity of HER2 inhibitors[23], [24].The mTOR pathway is also related to endocrine therapy resistance. SERM-resistant MCF-7/HER2 cells up-regulate mTOR expression by activating the PI3K/AKT, ERK and MAPK signaling pathways. The activated Phosphoinositide 3-kinase (PI3K)/AKT pathway stimulates mTOR to phosphorylate its downstream effectors p70 ribosomal S6 kinase (p70S6K) and eukaryotic initiation factor 4E binding protein 1 (4EBP1), mediating the expression of genes associated with tumor malignancy[25], [26]. Therefore, the combination of mTOR inhibitors and hormone- or HER2-targeting therapies was believed to be a promising strategy for overcoming initial therapeutic resistance and for preventing the development of resistance in ER+/HER2+ breast cancer[26].There is an intimate relationship between FASN and mTOR. mTOR activation induces FASN expression, and inhibiting the mTOR signaling pathway down-regulates FASN expression. FASN inhibition up-regulates DDIT4, a negative regulator of the mTOR pathway, suggesting that FASN inhibition negatively regulates the mTOR pathway via DDIT4[27], [28].

In this study, we found that FASN was overexpressed in ER+/HER2+ breast cancer cells. And the transcriptional activity of FASN promoter was high in ER+/HER2+ breast cancer cells. The crosstalk between ER and HER2 activated mTOR through the PI3K/AKT pathway and then promoted the expression of FASN, contributing to malignant transformation in ER+/HER2+ breast cancer cells. Inhibiting mTOR could down-regulate FASN expression. A FASN inhibitor and an mTOR inhibitor synergized to diminish the malignant phenotype of ER+/HER2+ breast cancer cells. These findings demonstrated that inhibiting the mTOR-FASN axis represents a promising new strategy for treating ER+/HER2+ breast cancer.

## Materials and Methods

### Cells, plasmids, reagents and antibodies

MCF-7(ER+), SKBR3 (HER2+)and MCF-7/HER2(ER+/HER2+, MCF-7 cell linestably transfected with a HER2 overexpression vector) breast cancer cells were obtained from the Department of Medical Genetics and Developmental Biology, Fourth Military Medical University, China. The cells were routinely cultured in Dulbecco's Modified EagleMedium (DMEM, Invitrogen) containing 10% heat-inactivated fetal bovine serum (Hyclone), 1% L-glutamine, 50 U/mL penicillin and 50 µg/mL streptomycin(Gibco)in a humidified incubator at 37°C in a5% CO_2_ atmosphere. The recombinant luciferase vector pGL3-FASN, which contains the 680-bp human FASN promoter in the promoter I region, was constructed in our lab and has been previously described [29]. The purified goat polyclonal anti-human FASN and mouse monoclonal anti-human GAPDH antibodies were purchased from Santa Cruz Biotechnology. Horseradish peroxidase (HRP)-conjugated anti-goat IgG and anti-mouse IgG secondary antibodies were purchased from Sigma-Aldrich. The anti-AKT, anti-phospho-AKT (Ser473), anti-mTOR, anti-phospho-mTOR(Ser2448), anti-phospho-p70S6K (Thr421/Ser424), anti-phospho-p70S6K (Thr389) and anti-phospho-4EBP1 (Ser65)antibodies were obtained from Cell Signaling Technology. The PI3K inhibitor LY294002and the mTOR inhibitor rapamycin were purchased from Calbiochem (EMD, Millipore), and the FASN inhibitor ceruleninwas obtained from Sigma-Aldrich. For the *in vivo* studies, cerulenin was dissolved in 25% dimethyl sulfoxide(DMSO, Sigma-Aldrich), and rapamycin was dissolved in 2% ethyl alcohol.

### Western blotting

The cells were scraped and lysed after being treated with pathway inhibitors. Equivalent amounts of supernatant containing the solubilized proteins were separated by sodium dodecyl sulfate polyacrylamide gel electrophoresis (SDS/PAGE) on 6% gels (for FASN) or 12% gels (for other proteins) and were transferred to PVDF membranes (Bio-Rad). The PVDF membranes were blocked for 1 h in blocking buffer [2% non-fat milk and 0.05% Tween-20 in Tris-buffered saline (TBS)] at room temperature (RT). The membranes were incubated overnight at 4°C with the antibodies diluted in blocking buffer (1∶100) and subsequently washed with TBS containing 0.05% Tween-20 at RT. The membranes were incubated for 1 h with secondary antibody (1∶1000dilution in blocking buffer) at RT. The western blotting kit was purchased from Roche Biotech. After three washes, the membranes were exposed to HRP for 5 min. The membranes were exposed to autoradiography X-ray films for between 10sec and 30 min. The X-ray films were scanned.

### Indirect immunofluorescence

Breast cancer cells grown on tissue culture chamber slides were rinsed with phosphate-buffered saline (PBS) and then fixed in 4% paraformaldehyde for 20 min. The cells were washed three times with PBS, blocked with blocking buffer (1%BSA and 0.3% Triton X-100 in PBS) for 30 min and then incubated with the goat polyclonal anti-human FASN antibody (1∶100 dilution in blocking buffer) for 1 hat 37°C. After extensive washing, the cells were incubated for 45 min with Cy3-conjugated anti-goat IgG secondary antibody(1∶50 dilution in blocking buffer) at 37°C. After washing, the slides were placed in mounting media. As a control, cells were stained with secondary antibody alone. The experiment also settled negative control. The cells were then examined using a fluorescence microscope.

### Luciferase assay

SKBR3, MCF-7and MCF-7/HER2 cells(5×10^5^ per well) were seeded in 6-well plates16–20 h before transfection such that the cells were 70–80%confluent at the time of transfection. The cells were transiently transfected with pGL3-FASNusing Lipofectamine 2000 (Life Technologies). The pGL3 Luciferase Reporter Vector system was purchased from Promega.pGL3-control (driven by an SV40 promoter) and pGL3-TK (no promoter) were used as the positive and negative controls, respectively. pSV-β-gal was co-transfected with the test plasmids in each experiment as an internal control. Forty-four hours after transfection, the cells were treated with 2.5 nmol/L rapamycin for 4 h and then lysed with 100 µL of cell lysis buffer (91 mmol/L K_2_HPO_4_, 9 mmol/L KH_2_PO_4_, 100 mL/L glycerol, 1 mmol/L DTT and 100 mL/L Triton X-100); the luciferase activity of the cells was determined using the Luciferase Reporter Assay System (Promega). The relative luciferase activity of the reporter plasmid was normalized to pSV-β-gal activity, which was measured as the absorption at A420. The transcriptional activity of each plasmid was expressed as a percentage of the SV40 promoter-mediated activity. All of the values are presented as the mean of three independent experiments.

### RNA extraction and RT-PCR

Total RNA was isolated using TRIzol (Invitrogen), and cDNA was synthesized using 2 to 5 µg of total RNA as the template. The resulting cDNA was used as the template for RT-PCR. The relative gene expression was calculated based on β-actin gene expression. Human FASN (NM_004104.4) was amplified using the following primers: forward, 5′-CATCCAGATAGGCCTCATAGAC-3; and reverse, 5′-CTCCA TGAAGTAGGAGTGGAAG-3. β-actin was used as an internal control and was amplified using the following primers: forward, 5′-AGAAGGAGATCACTGC CCTGGCACC-3′; and reverse, 5′-CCTGCTTGCTGATCCACATCTGCTG-3′. The FASN PCR conditions were as follows: 95°C for 1 min and 40 cycles of 95°C for 20 s, 60°C for 20 s, 70°C for 15 s and 80°C for 10 s.

### Cell viability assay

SKBR3, MCF-7 and MCF-7/HER2 cells were plated in 96-well plates in triplicate data density of 3,000 cells per well. After 16–20 h, the medium was removed, and the cells were treated with cerulenin(0,2.5,5,7.5 or 10 mg/L), rapamycin(0,2.5,5 or 10 nmol/L) or the combination (5 mg/L cerulenin +2.5 nmol/L rapamycin) for 24 h. 3-(4,5-Dimethylthiazol-2-yl)-2,5-diphenyltetrazolium bromide (MTT, Sigma-Aldrich) assays were used to evaluate cell survival. Untreated control cells were cultured under the same conditions. The data are presented as the mean of three independent experiments performed in triplicate.

### Cell migration

Cell migration was evaluated using an 8-µm pore size transwell system (Corning). Cells that were serum-starved overnight were pretreated with cerulenin(5 mg/L), rapamycin (2.5 nmol/L) or the combination (5 mg/L cerulenin and 2.5 nmol/L rapamycin)for 24 h, harvested by trypsinization and resuspended in serum-free DMEM. The top chamber of the transwell was filled with 2,000 cells in suspension, and 0.6 mL of DMEM containing 10% FBS was added to the bottom chamber. After incubation at 37°C in 5% CO_2_ for 6 h, the filters were removed, rinsed twice with 0.1 mmol/L PBS, fixed in 4% paraformaldehyde and stained with 600 µL of 1 mg/L4',6-diamidino-2-phenylindole, dihydrochloride (DAPI,Sigma-Aldrich) for 1 min. The cells on the upper side of the filter were removed using cotton swabs. The number of migrated cells on the lower side of the filter was quantitated by counting20 fields per transwell using an inverted widefield fluorescence microscope at 20X magnification. Each experimental condition was performed at least in duplicate.

### Apoptosis analysis

MCF-7/HER2cells were pretreated with cerulenin(2.5 mg/L), rapamycin (2.5 nmol/L) or the combination (2.5 mg/L cerulenin and 2.5 nmol/L rapamycin) for 12 h. The cells were washed twice with PBS and then were incubated in the dark for 15 min with binding buffer[10 mmol/L HEPES/NaOH (pH 7.4), 140 mmol/L NaCl and 2.5 mmol/L CaCl2], annexinV(200 g/L; BD Pharmingen) and propidium iodide (PI, 1 g/L; Sigma). The fluorescence of annexinV and PI was measured using FCM (flow cytometry, Epics Elite flow cytometer; Coulter). The data were analyzed using CellQuest.

### Tumor cell colony formation assays

To analyze *in vitro* tumorigenesis, MCF-7/HER2 cells were pretreated within 24-well plates (5×10^4^cells per well) in riplicate with cerulenin(5 mg/L), rapamycin (2.5 nmol/L) or the combination of cerulenin and rapamycin for 12 h. The treated cells were trypsinized, counted, diluted in fresh medium and seeded in 6-well plates at a density of 1,000 cells per well. The cells were incubated for 14 days to enable colony formation, after which they were fixed and stained with methylene blue in 50% ethanol. The number of colonies that contained at least 50 cells was counted in triplicate dishes.

### Animal study

The animal protocol was approved by the Fourth Military Medical University Animal Care and Use Committee. Female nude mice (purchased from the animal center of Fourth Military Medical University) aged 4 to 5 weeks received mammary fat pad injections of 5×10^6^ breast cancer cells. The mice were implanted with 17β-estradiol 60-day release tablets (Abcam) one day prior to tumor inoculation. Two weeks after the breast cancer cells were injected, mice with similar tumor masses were randomized into 4 treatment groups: control, vehicle (1% Tween-80 p.o. 5 days a week and 2% ethyl alcohol intraperitoneally (i.p.) twice a week), cerulenin (80 mg/kg/day i.p. in 0.2 mLof 25% DMSO for 7days), rapamycin (3 mg/kg i.p. twice a week) or the combination of cerulenin and rapamycin. The mice were treated for 4 weeks. The mouse weights and tumor sizes were measured twice weekly. The tumor volume was calculated according to the formula[1/2× length × width^2^]. The mice were sacrificed after the treatment period.

### Statistical analysis

All the experiments were performed in triplicate. The data are reported as the mean±SD. The statistical significance of the differences between the groups was determined by one-way analysis of variance (ANOVA) followed by Dunnett's test for multiple comparisons, unless otherwise stated. In all the comparisons, p≤0.05 was considered to be statistically significant. The statistical analyses were performed using SPSS version 17.0 (SPSS Inc., USA). Isobologram method was used for analyze the cytotoxity of cells treated with drugs. Interaction index (Ix) based on isobologram curve <1.0 was considered to have synergetic effect for cells treated with two drugs.

## Result

### FASN and mTOR expression in breast cancer cells

**Figure 1:**
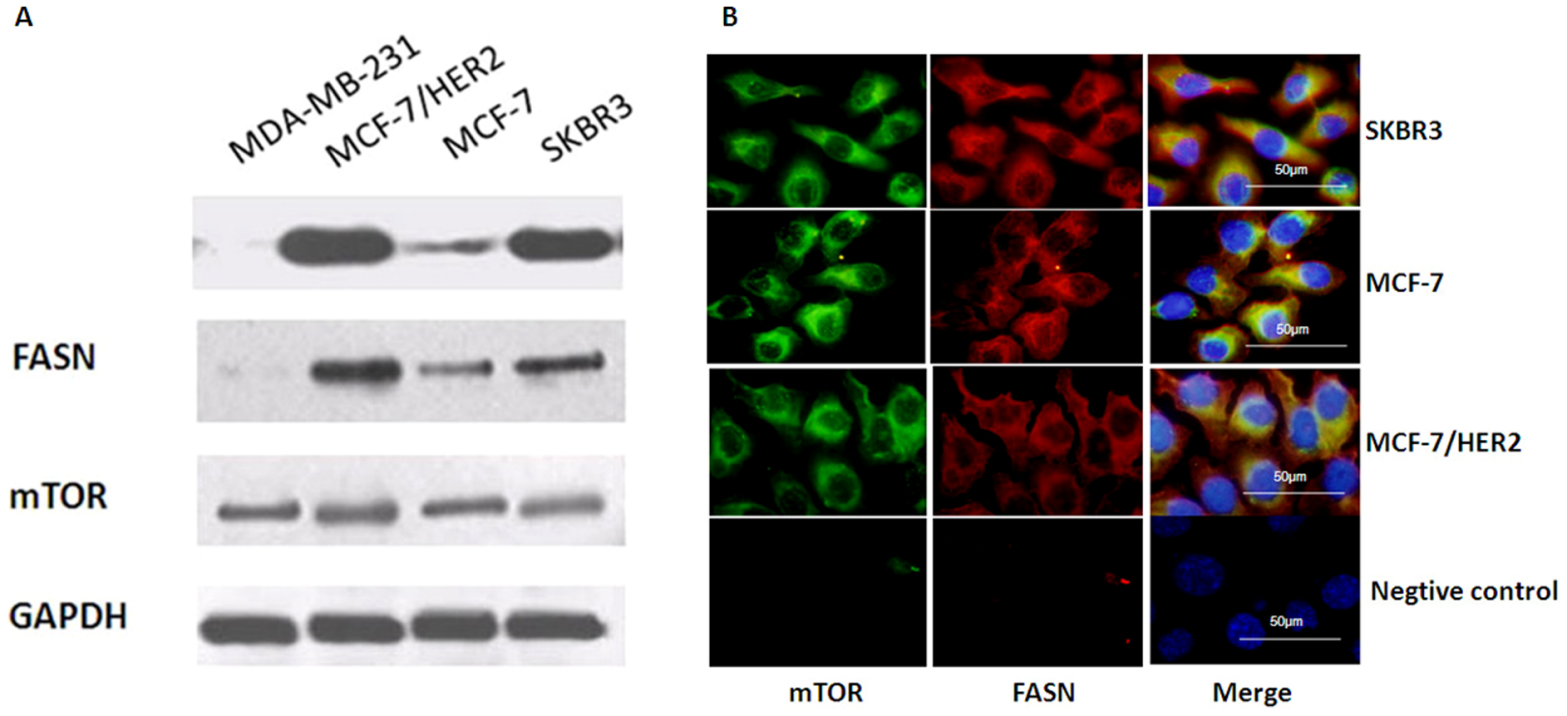
The expression of FASN and mTOR in SKBR3, MCF-7 and MCF-7/HER2 cells breast cancer cell lines. A, Western blotting indicated that FASN was expressed at different levels in all three breast cancer cell lines; FASN expression was higher in HER2-positive cells, such as SKBR3 and MCF-7/HER2 cells, than in MCF-7 cells, which expressed lower HER2 levels. FASN was not expressed in MDA-MB-231 breast cancer cells which showed negative HER2 expression. mTOR expression was similar in all four breast cancer cell lines. B, Indirect immunofluorescence analysis showed FASN and mTOR were localized primarily in the cytosol of cultured SKBR3, MCF-7 and MCF-7/HER2 cells.

We evaluated FASN and mTOR expression in breast cancer cell lines by western blotting and indirect immunofluorescence. Western blotting indicated that FASN was expressed at different levels in all three breast cancer cell lines; FASN expression was higher in HER2-positive cells, such as SKBR3 and MCF-7/HER2 cells, than in MCF-7 cells, which expressed lower HER2 levels. As a control, we also measured FASN expression in the ER/HER2-negative MDA-MB-231 breast cancer cell line and determined that FASN was not expressed. mTOR expression was similar in all four breast cancer cell lines (**Figure 1A**). This suggested that FASN expression was more closely associated with HER2 expression than with ER expression in breast cancer cells, whereas mTOR expression was independent of HER2 and ER status. Using indirect immunofluorescence, we determined that FASN and mTOR were localized primarily in the cytosol of cultured SKBR3, MCF-7and MCF-7/HER2 cells (**Figure 1B**).

### The mTOR inhibitor rapamycin reduced the transcriptional activity of the FASN promoter and FASN mRNA expression in ER+/HER2+ breast cancer cells

**Figure 2:**
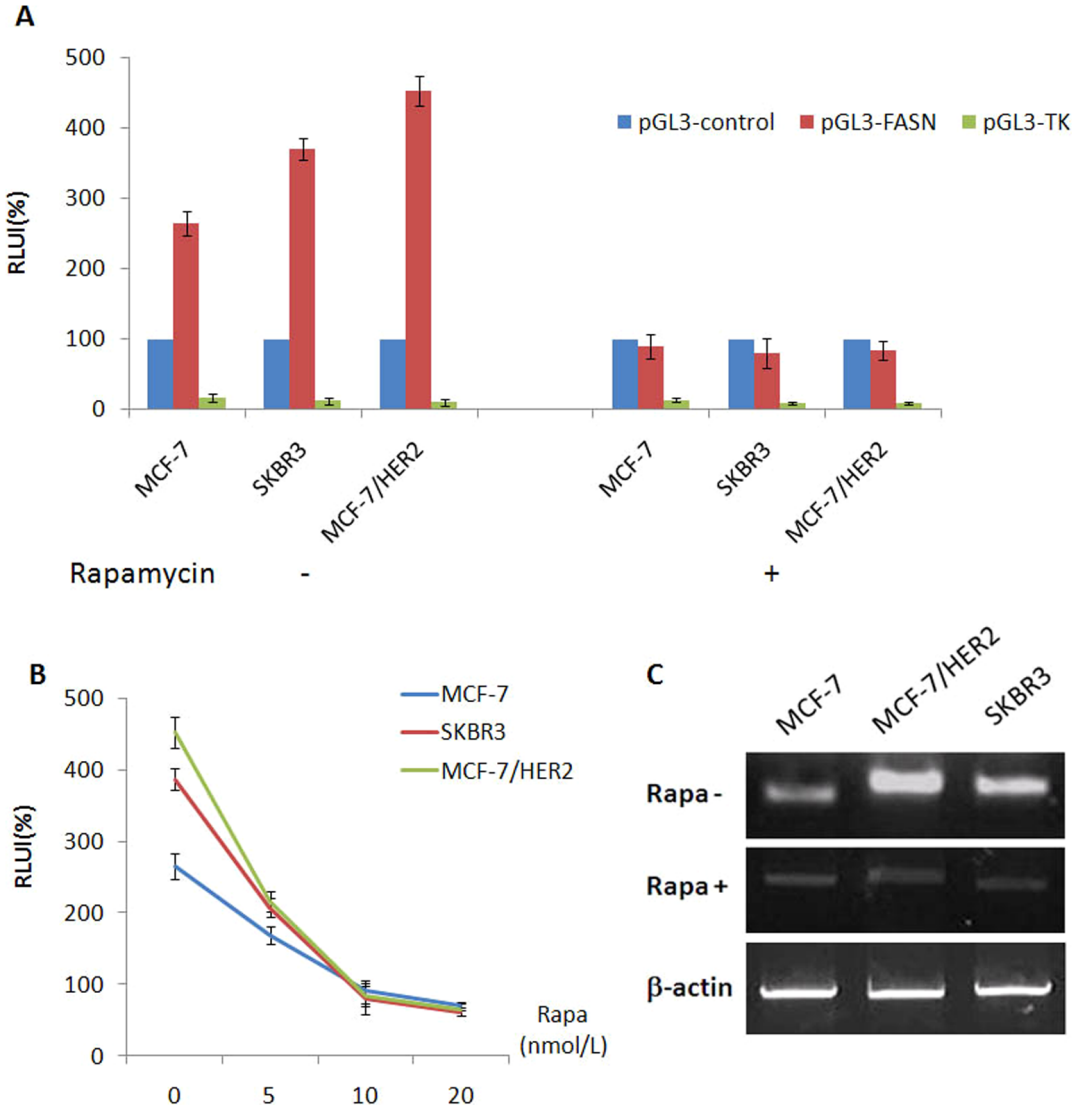
The transcriptional activities of FASN in SKBR3, MCF-7and MCF-7/HER2 breast cancer cells treated with mTOR inhibitor rapamycin. A, The transcriptional activities of FASN promoter in the three cells were all inhibited treated with rapamycin (2.5 nmol/L, 4 h) and the inhibition effect showed more significant in MCF/HER2 cell lines than in MCF-7 and SKBR3 cell lines (p<0.05). B, The transcriptional activities of FASN promoter in SKBR3, MCF-7and MCF-7/HER2 breast cancer cells treated with rapamycin at different concentration (0, 5, 10, 20 nmol/L, 4 h). The effect of rapamycin on the transcriptional activity of the FASN promoter was dose-dependent. C, FASN mRNA expression was higher in SKBR3 and MCF-7/HER2 cells than in MCF-7 cells. Rapamycin (2.5 nmol/L, 4 h) down-regulated FASN mRNA expression in all three breast cancer cell lines. Compared with MCF-7 cells, rapamycin-treated MCF-7/HER2 cells exhibited a significant decrease in FASN mRNA expression.

We used luciferase reporter assays to explore the effect of an mTOR inhibitor on FASN transcription in ER+/HER2+ breast cancer cells. We cloned the 680-bp human FASN Promoter I region from the human genome into the pGL3-enhance vector to construct a pGL3-FASN recombinant luciferase reporter vector; this vector has been previously shown to exhibit high transcriptional activity in FASN-overexpressing breast cancer cells [29]. We proved that the FASN promoter fragment exhibited high transcriptional activity in SKBR3, MCF-7 and MCF-7/HER2 cells. The transcriptional activity of the FASN promoter was significantly higher than that of the SV40 strong promoter (pGL3-control as the positive control) in all three breast cancer cell lines, and especially in SKBR3 and MCF-7/HER2 cells. The transcriptional activity of the FASN promoter in the three cell lines was inhibited by the mTOR inhibitor rapamycin (2.5 nmol/L, 4 h), and this inhibitory activity was more significant in MCF/HER2 cells than in MCF-7 or SKBR3 cells(p<0.05) (**Figure 2A**).The effect of rapamycin on the transcriptional activity of the FASN promoter was dose-dependent (**Figure 2B**). RT-PCR indicated that FASN mRNA expression was higher in SKBR3 and MCF-7/HER2 cells than in MCF-7 cells. Furthermore, rapamycin (2.5 nmol/L, 4 h) down-regulated FASN mRNA expression in all three breast cancer cell lines. Compared with MCF-7 cells, rapamycin-treated MCF-7/HER2 cells exhibited a significant decrease in FASN mRNA expression (**Figure 2C**).These results revealed that FASN mRNA expression and the transcriptional activity of the FASN promoter were closely linked to HER2 and mTOR. Inhibiting mTOR decreased FASN mRNA expression and the transcriptional activity of the FASN promoter.

### The PI3K/AKT/mTOR pathway was involved in regulating FASN expression in ER+/HER2+ breast cancer cells

**Figure 3:**
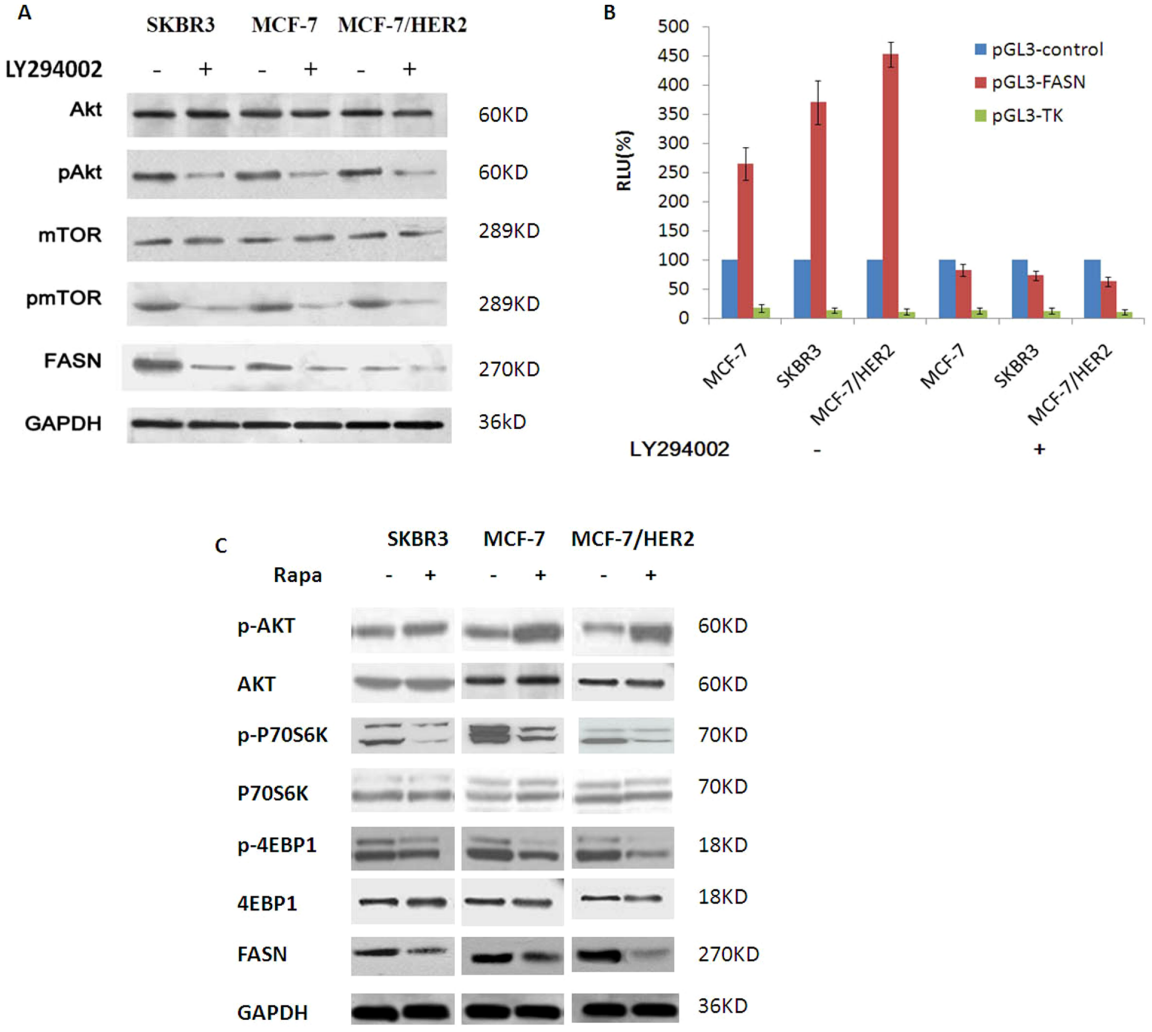
The PI3K/AKT/mTOR pathway was involved in regulating FASN expression in ER+/HER2+ breast cancer cells. A, PI3K inhibitor LY294002 (25 µmol/L, 12 h) could down-regulate the expression of pAKT, pmTOR and FASN but had no effect on the expression of total AKT or mTOR. B, The transcriptional activity of FASN promoter was obviously decreased in all the three breast cancer cell lines in response to LY294002(25 µmol/L, 12 h). The decrease in the transcriptional activity of the FASN promoter was most pronounced in MCF-7/HER2 cells (MCF-7/HER2 86.09%; SKBR3, 80.27%; and MCF-7, 69.05%). C, mTOR inhibitor rapamycin (20 nmol/L, 12 h) up-regulated the expression of pAKT but had no effect on total AKT levels. Rapamycin (20 nmol/L, 12 h) could up-regulate the expression of pAKT and down-regulate the expression of downstream targets of mTOR such as 4EBP-1 and pp70S6K and FASN, whereas the expression of 4EBP-1 and p70S6K did not change.

We used a PI3K/AKT pathway inhibitor and an mTOR inhibitor to probe the role of the PI3K/AKT/mTOR pathway on FASN expression in ER+/HER2+ breast cancer cells. The PI3Kinhibitor LY294002(25 µmol/L, 12 h) down-regulated the expression of pAKT, pmTOR and FASN but had no effect on the expression of total AKT or mTOR(**Figure 3A**). We further examined the transcriptional activity of the FASN promoter in the three breast cancer cell lines after treatment with LY294002 (25 µmol/L, 4 h). The transcriptional activity of the FASN promoter decreased in all three breast cancer cell lines in response to LY294002. The decrease in the transcriptional activity of the FASN promoter was most pronounced in MCF-7/HER2 cells (MCF-7/HER2 86.09%; SKBR3, 80.27%; and MCF-7, 69.05%)(**Figure3B**). Next, we determined that the mTOR inhibitor rapamycin (20 nmol/L, 12 h) up-regulated the expression of pAKT but had no effect on total AKT levels. Rapamycin down-regulated the expression of downstream targets of mTOR, such as p4EBP-1, pp70S6K and FASN, whereas the expression of 4EBP-1 and p70S6K did not change (**Figure 3C**).Therefore, our results suggested that the transcriptional activity of the FASN promoter and the expression of FASN in breast cancer cells were regulated by the PI3K/AKT signaling pathway, mediated by mTOR family members. The inhibition of these signaling pathways down-regulated the transcriptional activity of the FASN promoter and FASN overexpression; this inhibition was more significant in ER/HER2-positive breast cancer cells.

### A FASN inhibitor and an mTOR inhibitor synergized to induce cytotoxicity in breast cancer cells

**Figure 4:**
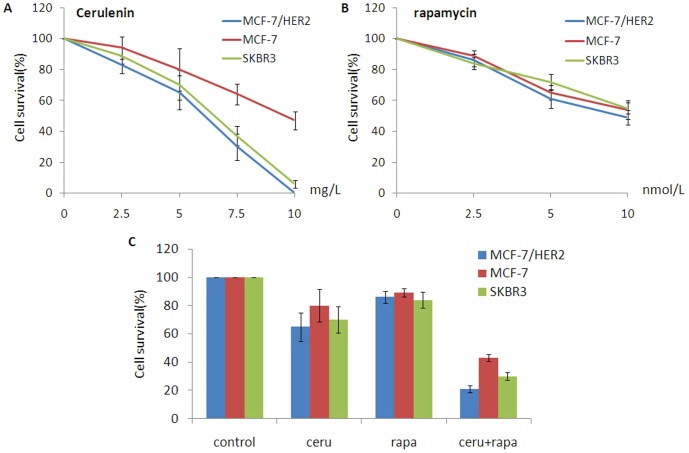
The cytotoxic effect of FASN inhibitor cerulenin and mTOR inhibitor rapamycin on SKBR3, MCF-7and MCF-7/HER2 breast cancer cells. A, Cerulenin (0, 2.5, 5, 7.5, 10 mg/L) could develop cytotoxic effect on all the three breast cancer cells, and the cytotoxic effect was more significant in HER2-overexpressing cells (SKBR3 and MCF-7/HER2). Cerulenin induced100% cytotoxicity at a concentration of 10 mg/L (24 h treatment) in SKBR3 and MCF-7/HER2 cells, whereas 47% of MCF-7 cells survived under the same condition (p<0.01). B, Rapamycin (0, 2.5, 5,10 nmol/L) was also cytotoxic in all three cell lines but did not exhibit a significant difference in cytotoxicity between the cell lines (p>0.05). C, The combination of rapamycin and cerulenin induced synergistic cytotoxicity in all three breast cancer cell lines, particularly in the ER+/HER2+ cells(MCF-7/HER2>SKBR3>MCF-7, p<0.05). The cytotoxicity of the rapamycin/cerulenin combination was higher than that of cerulenin or rapamycin alone in SKBR3 and MCF-7/HER2 breast cancer cells (p<0.05). In MCF-7 cells, the cytotoxicity of the rapamycin/cerulenin combination was not significantly different than cerulenin alone (p>0.05), but this combination was more cytotoxic than rapamycin alone (p<0.05). There were no differences between rapamycin and cerulenin in any of the three cell lines (p>0.05). In MCF-7/HER2 cells, a low dose of rapamycin (2.5 nmol/L) combined with a low dose of cerulenin (5 mg/L) induced significant cytotoxicity.

**Figure 5:**
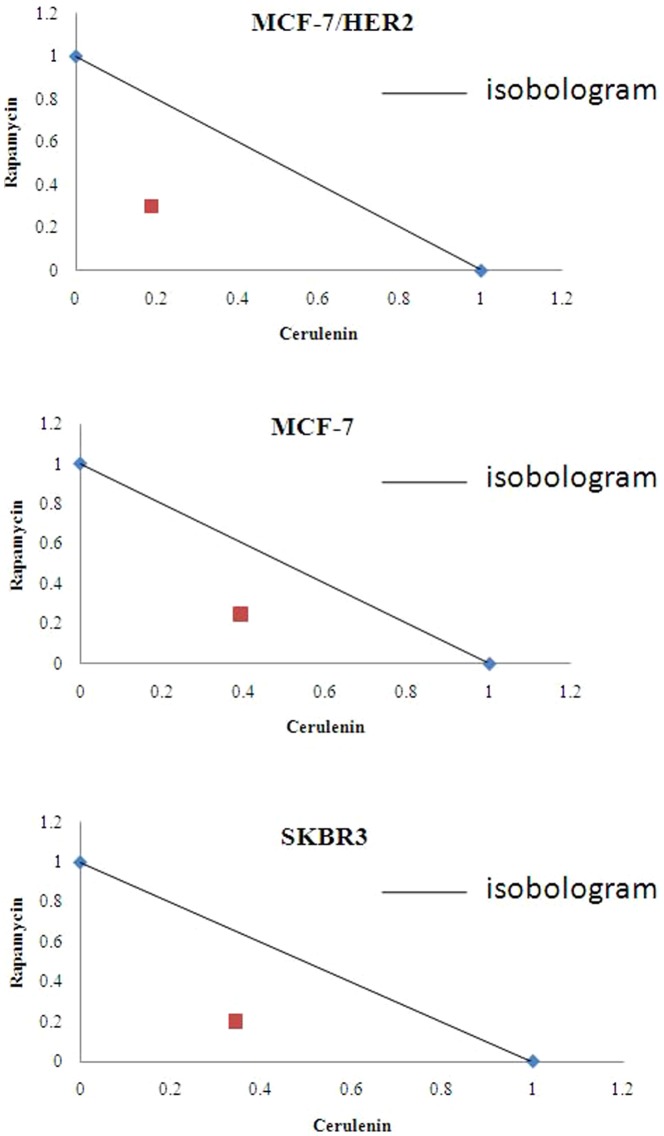
Interaction index (Ix) based on isobologram curve for MCF-7, SKBR3 and MCF-7/HER2 breast cancer cells with cerulenin combined with rapamycin treatment showed the combination treatment could produce synergetic cytotoxicity. In MCF-7/HER2 cells, the IC50 was 1.85 and the Ix was 0.4888. In MCF-7 cells, the IC50 was 3.06 and the Ix was 0.6389. In SKBR3 cells, the IC50 was 2.25 and the Ix was 0.5451.

**Figure 6:**
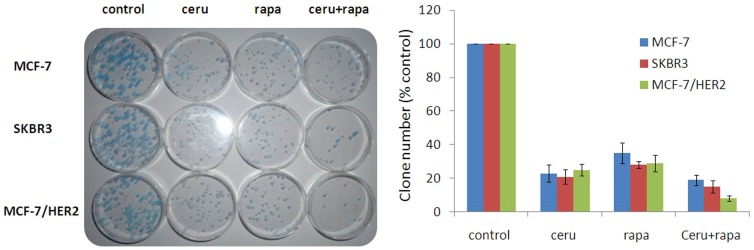
Tumor Cell Clone Formation Assays. The inhibition of colony formation was highest in cells treated with both rapamycin (5 nmol/L) and cerulenin (5 mg/L). The combination treatment inhibited colony formation by 92%; this effect was greater than that of cerulenin or rapamycin alone (75% and 71%, respectively; p<0.05), and the single-agent groups exhibited no obvious differences in ER+/HER2+ cells. The inhibition of colony formation by the combination was more pronounced in the MCF-7/HER2 cell line (MCF-7/HER2>SKBR3>MCF-7).

The FASN inhibitor cerulenin and the mTOR inhibitor rapamycin each cause cell death, as shown in previous studies[26], [27].To determine whether the mTOR inhibitor synergized with the FASN inhibitor to induce cytotoxicity, the breast cancer cells were treated with cerulenin, rapamycin or the combination of cerulenin and rapamycin. Cerulenin was cytotoxic in all three breast cancer cell lines, and the cytotoxicity was more significant in HER2-overexpressing cells (SKBR3 and MCF-7/HER2)(**Figure 4A**). Cerulenin induced100% cytotoxicity at a concentration of 10 mg/L (24 h treatment) in SKBR3 and MCF-7/HER2 cells, whereas 47% of MCF-7 cells survived under the same condition (p<0.01). Rapamycin was also cytotoxic in all three cell lines but did not exhibit a significant difference in cytotoxicity between the cell lines (p>0.05)(**Figure 4B**).The combination of rapamycin and cerulenin induced synergistic cytotoxicity in all three breast cancer cell lines, particularly in the ER+/HER2+ cells(MCF-7/HER2>SKBR3>MCF-7, p<0.05). The cytotoxicity of the rapamycin/cerulenin combination was higher than that of cerulenin or rapamycin alone in SKBR3and MCF-7/HER2 breast cancer cells (p<0.05). In MCF-7 cells, the cytotoxicity of the rapamycin/cerulenin combination was not significantly different than cerulenin alone (p>0.05), but this combination was more cytotoxic than rapamycin alone (p<0.05). There were no differences between rapamycin and cerulenin in any of the three cell lines (p>0.05). In MCF-7/HER2 cells, a low dose of rapamycin (2.5 nmol/L) combined with a low dose of cerulenin (5 mg/L) induced significant cytotoxicity **(** **Figure 4C**). Interaction index (Ix) based on isobologram curve [30], [31](for MCF-7, SKBR3 and MCF-7/HER2 breast cancer cells with cerulenin combined with rapamycin treatment showed the combination treatment could produce synergetic cytotoxicity (**Figure 5**). Tumor cell colony formation assays were used to determine the tumorigenic potential of these breast cancer cell lines. The data obtained from this assay correlated well with the MTT assay results. The inhibition of colony formation was highest in cells treated with both rapamycin (5 nmol/L) and cerulenin(5 mg/L). The combination treatment inhibited colony formation by 92%; this effect was greater than that of cerulenin or rapamycin alone (75% and 71%, respectively; p<0.05), and the single-agent groups exhibited no obvious differences in ER+/HER2+ cells. The inhibition of colony formation by the combination was more pronounced in the MCF-7/HER2 cell line (MCF-7/HER2>SKBR3>MCF-7) (**Figure 6**).

### A FASN inhibitor synergized with an mTOR inhibitor to induce apoptosis and inhibit cell migration in ER+/HER2+ breast cancer cells

**Figure 7:**
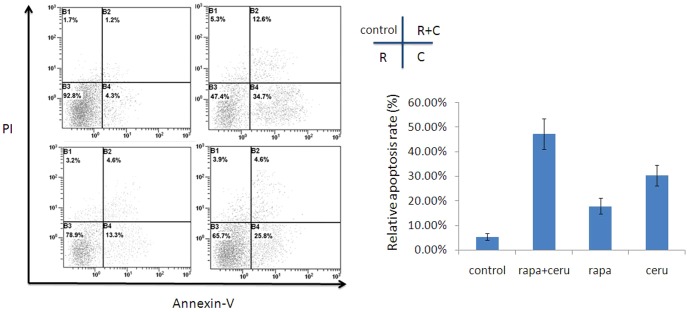
FASN inhibitor cerulenin combined with mTOR inhibitor rapamycin could synergistically induced apoptosis in MCF-7/HER2 breast cancer cells. Apoptosis was measured through FACS analysis of PI or Annexin-V stained cells 12 h after treatment with cerulenin (2.5 mg/L) and rapamycin (2.5 nmol/L). Column data analysis of intact cells (Annexin-V–/PI–), early apoptotic(Annexin-V+/PI–) and late apoptotic (Annexin-V+/PI+) for each cell group. The early apoptotic rate in MCF-7/HER2 cells treated with rapamycin and cerulenin was 34.7% higher than that in individually treated cells (rapamycin 13.3%, p<0.01; cerulenin 25.8%, p>0.05). The early and late apoptoticfractions in the combination-treated cells totaled 47.3%, which was significantly higher than the fraction in rapamycin-treated cells (21.6%, p<0.05) but did not differ from cerulenin-treated cells (47.3% vs. 30.4%, p>0.05).

**Figure 8:**
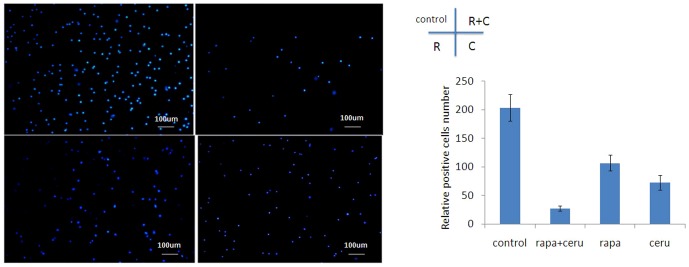
Rapamycin could induce systemic anti-migration effect combined with cerulenin determined with Transwell migration assay in MCF-7/HER2 cells. Compared with the control, rapamycin (2.5 nmol/L) combined with cerulenin (5 mg/L) inhibited cell migration by 86.28%, whereas rapamycin and cerulenin individually inhibited cell migration by 47.55% and 64.22%, respectively. Migration was significantly different between the combination treatment group and the rapamycin group (p<0.05),but there were no differences in migration between the combination group and the cerulenin group (p>0.05).

We further explored whether cerulenin synergized with rapamycin to induce apoptosis in ER+/HER2+ breast cancer cells. We performed annexinV FACS (fluorescence-activated cell sorting) analysis to analyze apoptosis after treatment with cerulenin (2.5 mg/L), rapamycin (2.5 nmol/L) or the rapamycin/cerulenin combination. The early apoptotic rate in MCF-7/HER2 cells treated with rapamycin and cerulenin was 34.7% higher than that in individually treated cells (rapamycin 13.3%, p<0.01; cerulenin 25.8%, p>0.05). The early and late apoptoticfractions in the combination-treated cells totaled 47.3%, which was significantly higher than the fraction in rapamycin-treated cells (21.6%, p<0.05) but did not differ from cerulenin-treated cells (47.3% vs. 30.4%, p>0.05)(**Figure 7**). We also examined the ability of the combination treatment to inhibit the migration of MCF-7/HER2 cells using a transwell system. Compared with the control, rapamycin (2.5 nmol/L) combined with cerulenin (5 mg/L) inhibited cell migration by 86.28%, whereas rapamycin and cerulenin individually inhibited cell migration by 47.55% and 64.22%, respectively. Migration was significantly different between the combination treatment group and the rapamycin group (p<0.05),but there were no differences in migration between the combination group and the cerulenin group (p>0.05)(**Figure 8**).

### AFASN inhibitor and an mTOR inhibitor synergized to inhibit tumorigenesis *in vivo*

**Figure 9:**
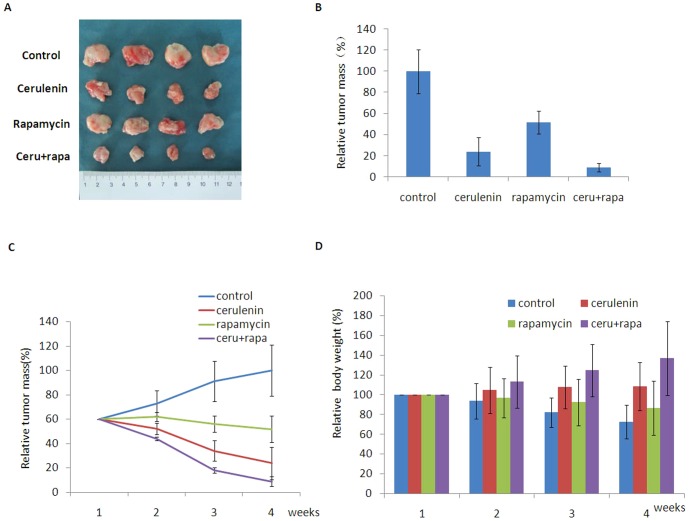
Animal study showed that cerulenin combined with rapamycin could mediate synergistic anti-tumor effect in mice incubated with MCF-7/HER2 breast cancer cells. A and B, Cerulenin combined with rapamycin elicited approximately 91% tumor regression compared with the negative control, whereas rapamycin and cerulenin resulted in 76% and 48% tumor regression, respectively. Tumor regression was significantly different in the combination and the rapamycin-only groups (p<0.05). C, The realtive time/tumor mass of mice after treating with cerulenin combined with rapamycin. This anti-tumorigenic effect of the combination treatment was time-dependent. D, Tumor regression was accompanied by an increase in body weight. Compared with baseline, the body weight of the mice increased by 36.5% in the combination group and by 8.2% in the cerulenin-only group, whereas the body weight decreased by 27.5% and 13.6% in the negative control and rapamycin-only groups, respectively.

We examined the effect of the combination of the FASN and mTOR inhibitors on tumorigenesis in nude mice inoculated with MCF-7/HER2 cells. Cerulenin combined with rapamycin elicited approximately 91% tumor regression compared with the negative control, whereas rapamycin and cerulenin resulted in 76% and 48% tumor regression, respectively (**Figure 9A, B**). Tumor regression was significantly different in the combination and the rapamycin-only groups (p<0.05). This anti-tumorigenic effect of the combination treatment was time-dependent (**Figure 9C**). Tumor regression was accompanied by an increase in body weight. Compared with baseline, the body weight of the mice increased by 36.5% in the combination group and by 8.2% in the cerulenin-only group, whereas the body weight decreased by 27.5% and 13.6% in the negative control and rapamycin-only groups, respectively (**Figure 9D**). These results indicated that the FASN and mTOR inhibitors synergized to inhibit tumor growth in MCF-7/HER2 xenografts.

## Discussion

The crosstalk between ER and HER2 is the primary mediator of the malignant phenotype in ER+/HER2+ breast cancer. Conventional SERMs and HER2 inhibitors do not extend OS in patients with this complex subtype of breast cancer. Therefore, interfering with growth factor-driven signaling pathways and downstream effectors implicated in ER/HER2 crosstalk may represent potential new strategies for the treatment of ER+/HER2+ breast cancer.

In this study, FASN was identified as the key downstream effector in ER/HER2 crosstalk in ER+/HER2+ breast cancer cells. FASN was more highly expressed and had a more active promoter in ER/HER2-positive breast cancer cells than in breast cancer cells positive for only ER or HER2. Therefore, inhibiting FASN represents a promising anticancer strategy in ER+/HER2+breast cancer. Although there are several novel FASN inhibitors [16], [32], cerulenin and C75 are the most utilized FASN inhibitors, and they exhibit potent anti-tumor activity in cancer cells[18]. Unfortunately, these compounds have been shown to affect nutrient uptake and body weight in mice, which hampers their clinical application [33]. It is thought that blocking key signaling pathways upstream of FASN might decrease the required dose of conventional FASN inhibitors and enhance antitumor activity.

In our study, the mTOR signaling pathway was determined to connect ER/HER2 crosstalk with the downstream effector FASN. We found that the mTOR inhibitor rapamycin inhibited the transcriptional activation of the FASN promoter and FASN mRNA expression in breast cancer cells, especially in ER+/HER2+ breast cancer cells. Further studies revealed that the PI3K/AKT/mTOR pathway regulated FASN expression. The PI3K inhibitor LY294002 and rapamycin inhibited FASN expression. Activated mTOR, a positive regulator of cell proliferation, stimulates protein translation and ribosome biogenesis by phosphorylating the key translation regulators p70S6K and 4EBP1, resulting in the expression of critical cell proliferation proteins and promoting the G1/S cell cycle transition[22]. The phosphorylation of 4EBP1 and p70S6K, key substrates of mTOR signaling, were up-regulated by the mTOR inhibitor. p4EBP1 and p70S6K are critical for cell proliferation[22], [34], [35]. p4EBP1 is primarily expressed in poorly differentiated tumors and correlates with tumor size, the presence of lymph node metastasis and loco-regional recurrence[36]-[37]. Similarly, p70S6K overexpression is associated with aggressive disease and a poor prognosis in breast cancer. mTOR/p70S6K1 activation is associated with acquired resistance to HER2-TKI. In lapatinib-refractory MCF-7/HER2 cells, the HER2 signaling pathway remains largely intact, and the cells exhibit a dramatic hyperphosphorylation of p70S6K1[38], [39].

Inhibiting mTOR signaling was presumed to enhance the antitumor activity of the FASN inhibitor. Large studies have been performed to test mTOR inhibitors against cancer.The combination of mTOR inhibitors with hormone- or HER2-targeted therapies appears to be a promising strategy for overcoming resistant disease and preventing the development of resistance[40]–[41]. The systemic antitumor effect of an mTOR inhibitor may be due to interference with ER and HER2. In this study, we investigated the role of a FASN inhibitor combined with an mTOR inhibitor in cancer cell growth. Our results showed that the combination of the mTOR and FASN inhibitors potently inhibited the growth and invasive capacity of three breast cancer cell lines, even at low concentrations. ER+/HER2+ cells were more sensitive to this combination treatment than the other cell lines, indicating that the ER/HER2 crosstalk in dual-positive breast cancer cells was more dependent on mTOR-FASN signaling. Animal studies confirmed that the combination of the FASN and mTOR inhibitors inhibited the progression of ER+/HER2+ breast cancer xenografts. Notably, the FASN inhibitor in combination with the mTOR inhibitor caused tumor regression and induced weight gain in mice bearing ER+/HER2+ tumors.

In conclusion, our study showed that ER/HER2 crosstalk activated mTOR through upstream cell signaling pathways. Furthermore, the activated mTOR pathway promoted FASN expression, which results in the malignant phenotype transformation of breast cancer cells. Inhibiting the mTOR-FASN axis represents a promising new strategy for treating ER/HER2-positive breast cancer; the studied combination treatment exhibited potent anti-tumor activity that may overcome resistance to endocrine therapy and HER2 inhibitors and avoid the side effects of high doses of FASN inhibitors.
